# Protein intake from different sources and cognitive decline over 9 years in community-dwelling older adults

**DOI:** 10.3389/fpubh.2022.1016016

**Published:** 2022-10-14

**Authors:** Rongtao Gao, Zhan Yang, Wenju Yan, Weiping Du, Yuan Zhou, Feng Zhu

**Affiliations:** ^1^Department of Chronic Disease Control and Prevention, Tai'an Center for Disease Control and Prevention, Tai'an, China; ^2^Tai'an Maternal and Child Health Care Hospital, Tai'an, China; ^3^Department of Vascular Cardiology, Tai'an Central Hospital, Tai'an, China; ^4^Department of Hematology, Second Affiliated Hospital of Shandong First Medical University, Tai'an, China

**Keywords:** dietary protein, animal protein, plant protein, cognitive decline, protein intake

## Abstract

**Objectives:**

To examine the association of protein intake from different sources with cognitive decline.

**Methods:**

Our analysis included 3,083 participants aged 55–93 years from the China Health and Nutrition Survey. Cognition was assessed in 1997, 2000, 2004, 2006, and 2015. Diet intake was assessed using weighing methods in combination with 24-h dietary recalls for three consecutive days at each survey.

**Results:**

Participants consumed 13.94% of energy intake from total protein, with 11.47 and 2.47% from plant and animal sources, respectively. During a follow-up of 9 years, participants in quintile 5 of plant protein intake (% energy) had a higher risk [odds ratio (95% CI): 3.03 (1.22–7.53)] of cognitive decline compared with those in quintile 1. Higher animal protein intake (% total protein) was associated with a lower risk of cognitive decline [odds ratio (95% CI) for quintile 5 vs. quintile 1: 0.22 (0.07–0.71)]. Grains (plant source) protein intake was inversely but fish/shrimp and poultry (animal source) protein intake were positively associated with change in cognitive Z-score.

**Conclusion:**

Increasing animal protein consumption in a population with plant dominant diets may help to prevent cognitive decline.

## Introduction

The global number of individuals with dementia increased from 20.2 million in 1990 to 43.8 million in 2016 (1). Notably, China accounted for approximately one-quarter of the worldwide dementia population in 2016 (2). While the age-standardized prevalence of dementia worldwide increased by 1.7% from 1990 to 2016, in China, it increased by 5.6% during this same period (2). The epidemic of dementia and its subsequent cost impose a tremendous burden on economics and health system in China (3). This will become a more important concern with the increased aging population in China, therefore, it is imperative to target intervention priorities for the prevention of dementia and cognitive decline.

Diabetes, smoking, physical inactivity, and unhealthy dietary patterns have been identified as important modifiable risk factors for dementia (4, 5). Increasing evidence has shown that healthy dietary patterns are associated with a lower risk of cognitive decline and dementia, but the association between the intake of individual foods or nutrients and cognitive decline is inconsistent across studies (6–8).

Prospective studies investigating dietary patterns have highlighted the importance of foods rich in protein including grains, nuts, beans, fish, and poultry on the prevention of dementia (9, 10). Furthermore, animal and plant protein intakes have been shown to have divergent associations with well-known dementia risk factors including diabetes, hypertension, obesity, and metabolic syndrome (11, 12). This suggests the protein intake from different food sources may have different associations with cognitive decline. Several studies from Western countries with animal dominant diets have investigated the association between protein intakes from different sources and cognitive function with inconsistent findings (13, 14). However, no such data are available from Asian countries with plant dominant diets.

In this paper, we aimed to examine whether the protein intake from animal and plant foods and main food groups were predictive of cognitive decline. We also aimed to test the association between the composition of animal and plant proteins and cognitive decline.

## Materials and methods

### Participant selection

**Figure 1:**
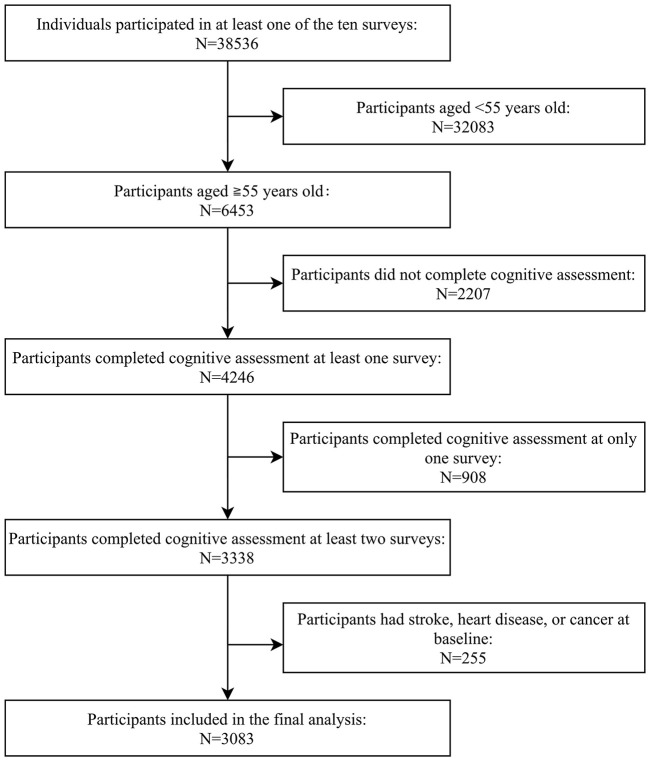
Flowchart for participant selection.

The China Health and Nutrition Survey (CHNS) is an ongoing open-cohort study initiated in 1989 and followed up in 1991, 1993, 1997, 2000, 2004, 2006, 2009, 2011, and 2015. The design and sampling have been detailed elsewhere (15, 16). Briefly, a multistage, random cluster process was used to select participants in nine provinces from northeast to southwest in China. Two cities and four counties were randomly selected in each province based on their income levels as reported by the State Statistical Bureau in 1988. Four communities in each city or county and 20 households in each community were then randomly selected. The response rate, based on those who participated in 1989, in the 2006 survey was >60%. Overall response rates, based on those who participated in at least two surveys, were around 88% at the individual level and 90% at the household level (15). Cognitive assessment in a sub-cohort of participants aged ≥55 years was conducted in 1997, 2000, 2004, 2006, and 2015. Of the 38,536 individuals who participated in any of the ten surveys, the following were excluded from the present analysis: those aged <55 years (*n* = 32,083), those who did not have cognitive function assessed (*n* = 2,207), those who completed the cognitive assessment at only one survey (*n* = 908), or those who had stroke, heart disease, or cancer at baseline (*n* = 255). A total of 3,083 participants were included in the final analysis (Figure 1).

The survey was approved by the institutional review committees of the University of North Carolina at Chapel Hill and the National Institute of Nutrition and Food Safety, Chinese Center for Disease Control and Prevention. Written informed consent was obtained from all participants.

### Dietary assessment

Dietary intake, at the household level and the individual level, was assessed using weighing methods in combination with 24-h dietary recalls for three consecutive days at each survey. All foods and condiments for the home food inventory at the beginning and end of the 3-day survey period were measured using scales and recorded by trained interviewers. Individual dietary data for the same three consecutive days were recorded for all household members and proportions of foods and condiments consumed at the household level were allocated to each individual (17). Food and nutrient intake at the individual level was then calculated.

Nutrients and energy intake was calculated based on the China Food Composition (18). We computed the protein intake from different sources separately by multiplying daily consumption by the protein content and summing this across foods/beverages. Foods were grouped as plant or animal sources and further broken down into grains, tubers, vegetables, fruits, beans, nuts (plant sources), red meat, poultry, fish/shrimp, dairy, and eggs (animal sources). The average annual protein intake from different sources of the surveys completed before the first cognitive assessment was also calculated.

The assessment of energy intake has been validated by using the doubly labeled water method with a correlation efficient of 0.56 for men and 0.60 for women (19).

### Cognitive function test

A subset of the items from the Telephone Interview for Cognitive Status–modified was used to assess cognitive function (20). The tool has been adopted in other population studies in China (21, 22). The cognitive screening included the immediate and delayed recall of a 10-word list, counting backward from 20, and serial seven subtraction from 100 for five times. Each correctly recalled word was assigned a score of 1 and the total score for immediate and delayed recall ranged from 0 to 20. For counting backward, a score of 2 was given to those counted backward correctly in the first try and one to those only counted backward correctly in the second try. A score of 1 was assigned to each of the 5 serial subtractions and the total score for serial seven subtraction ranged from 0 to 5.

The composite cognitive Z-score was computed by summing the scores of all three tasks and ranged from 0 to 27. The composite cognitive Z-score was analyzed in Z-score and a higher score represented better cognitive function. The change in composite cognitive Z-score was computed by subtracting the score at baseline from that at follow-up. Cognitive decline was defined as change in composite cognitive Z-score below the mean minus two standard deviations (SDs). This was retested by change in the composite cognitive Z-score below the mean minus 1.5 SDs.

### Physical examinations

Height was measured using a freestanding stadiometer and weight was measured using an electronic scale. Body mass index (BMI) was calculated based on weight and height, and overweight/obesity was defined as BMI ≥ 25 kg/m^2^ (23).

Blood pressure was measured using a standard mercury sphygmomanometer by trained nurses. Three measurements were taken to the nearest two mmHg and the average of the last two was used.

### Confounders

All confounders at the time of the first cognitive measure were used in the analysis. Demographic socioeconomic factors included age, gender, region, education, smoking, and alcohol consumption were collected using a questionnaire. Physical activity was assessed based on hours per week spent in different occupational, household, transportation, and leisure-time activities, from which metabolic equivalent of task (MET) was calculated (24). History of diabetes was also self-reported.

### Statistical analysis

Data were expressed as frequency (percentage) and means ± SDs. ANOVA for continuous variables and the Chi-square test for categorical variables was performed to compare the difference of baseline characteristics across the quintiles of protein intake.

Participants were divided into quintiles based on the % energy from protein from major food sources at baseline. General linear regression models were used to obtain coefficients for the change in composite cognitive Z-score for quintiles 2–5 vs. the quintile 1 and per 1% increment in energy intake from animal foods, plant foods, grains, tubers, vegetables, fruits, beans, nuts, red meat, poultry, fish, dairy, and eggs. The following models were tested: (1) age and gender; (2) model 1 plus education, urbanization, duration of follow-up, smoking, alcohol intake, physical activity, composite cognitive Z-score, diabetes, BMI, systolic, and diastolic blood pressure at baseline; (3) model 2 plus intake of energy, sodium, potassium, fat, and fiber. We also calculated the linear trend by assigning participants the median intake within each quintile of the percentage of energy from dietary protein for each food source. Logistic regression models were used to examine whether protein intakes from different food sources were associated with cognitive decline.

Whether the composition of animal and plant protein intake (% total protein) was predictive of the change in composite cognitive Z-score and cognitive decline was also examined where total protein intake was further adjusted for.

Moderation analysis was used to test whether the association between protein intake and cognitive decline depended on other important factors. Moderation analysis was conducted to examine whether the association of plant and animal protein intake with cognitive decline was modified by age, gender, education, urbanization, and follow-up duration.

Sensitivity analysis was conducted to examine whether the association of the average annual protein intake of surveys completed before the first cognitive assessment with changes in cognitive Z-scores.

Analyses were performed using SAS version 9.4 (SAS Institute Inc.) and all *P*-values were two-sided.

## Results

### Participant characteristics

A total of 3,083 participants (51.5% women) aged 55–93 (mean ± SD: 61.9 ± 6.6) years at baseline with complete data on variables of interest were included in the analysis. Individuals with a higher plant protein intake were more likely to have lower education, live in rural areas, currently smoke, and have higher physical and occupation activity levels compared to those with a lower plant protein intake. An inverse association between plant protein intake and composite cognitive Z-scores at baseline was observed (Tables 1, 2). In contrast, higher animal protein intake was associated with lower energy intake, lower fiber intake, and higher sodium intake. There was a positive association between the animal protein intake and the composite cognitive Z-score at baseline (Supplementary Table 1).

**Table 1:** Baseline characteristics across quintiles of plant protein intake.

	**Plant protein intake**					
	**Quintile 1**	**Quintile 2**	**Quintile 3**	**Quintile 4**	**Quintile 5**	*P*-value*
Age (years)	63.02 ± 6.97^†^	61.61 ± 6.51	61.48 ± 6.24	61.14 ± 6.08	62.00 ± 6.81	0.0026
Gender						0.44
Men	296 (48.0)^‡^	292 (47.4)	296 (48.1)	305 (49.4)	305 (49.4)	
Women	321 (52.0)	324 (52.6)	320 (51.9)	312 (50.6)	312 (50.6)	
Education						<0.0001
Illiterate	208 (33.7)	275 (44.6)	271 (44.0)	298 (48.3)	306 (49.6)	
Primary school	134 (21.7)	148 (24.0)	179 (29.1)	157 (25.4)	168 (27.2)	
Lower middle school	100 (16.2)	80 (13.0)	82 (13.3)	90 (14.6)	73 (11.8)	
Upper middle school	66 (10.7)	31 (5.0)	24 (3.9)	13 (2.1)	29 (4.7)	
Technical or vocational	54 (8.8)	31 (5.0)	24 (3.9)	23 (3.7)	17 (2.8)	
University or college	33 (5.4)	18 (2.9)	15 (2.4)	10 (1.6)	8 (1.3)	
Missing	22 (3.6)	33 (5.4)	21 (3.4)	26 (4.2)	16 (2.6)	
Living area						<0.0001
Urban	365 (59.2)	222 (36.0)	217 (35.2)	201 (32.6)	134 (21.7)	
Rural	252 (40.8)	394 (64.0)	399 (64.8)	416 (67.4)	483 (78.3)	
Smoking						0.0011
Never	436 (70.7)	430 (69.8)	410 (66.6)	402 (65.2)	389 (63.0)	
Former	21 (3.4)	14 (2.3)	18 (2.9)	24 (3.9)	24 (3.9)	
Current	160 (25.9)	172 (27.9)	188 (30.5)	191 (31.0)	204 (33.1)	
Alcohol intake						0.14
None	416 (67.4)	440 (71.4)	422 (68.5)	410 (66.5)	404 (65.5)	
Yes	201 (32.6)	176 (28.6)	194 (31.5)	207 (33.5)	213 (34.5)	
Diabetes						0.0316
No	592 (95.9)	593 (96.3)	600 (97.4)	603 (97.7)	602 (97.6)	
Yes	25 (4.1)	23 (3.7)	16 (2.6)	14 (2.3)	15 (2.4)	
Composite cognitive Z-score	0.24 ± 0.84	0.13 ± 0.92	0.11 ± 0.89	0.02 ± 0.91	0.02 ± 0.94	<0.0001
Memory Z-score	10.26 ± 4.25	10.14 ± 4.50	9.97 ± 4.30	9.47 ± 4.49	9.41 ± 4.37	<0.0001
Subtraction Z-score	3.75 ± 1.74	3.53 ± 1.85	3.62 ± 1.84	3.70 ± 1.79	3.52 ± 1.91	0.20
Backward counting Z-score	1.03 ± 0.44	1.02 ± 0.50	1.02 ± 0.47	0.98 ± 0.48	0.99 ± 0.48	0.0856
Physical activity (MET-h/wk)	136.1 ± 106.7	195.5 ± 119.7	194.2 ± 118.4	200.9 ± 117.7	219.3 ± 114.8	<0.0001
BMI (kg/m^2^)	23.66 ± 3.51	23.21 ± 3.61	23.25 ± 3.60	22.77 ± 3.56	22.51 ± 3.38	<0.0001
Diastolic blood pressure (mmHg)	82.15 ± 10.82	81.43 ± 11.39	81.12 ± 11.91	80.93 ± 11.66	80.45 ± 12.12	0.0082
Systolic blood pressure (mmHg)	130.4 ± 19.3	128.9 ± 19.0	128.9 ± 19.6	129.8 ± 20.5	127.7 ± 20.3	0.0797

^*^ANOVA was used to test the difference of continuous variables across quintiles of plant protein intake and Chi-square for categorical variables.

^†^All such data were means ± standard deviations.

^‡^All such data were frequency (percentage).

**Table 2:** Dietary intakes across quintiles of plant protein intake.

	**Plant protein intake**					
	**Quintile 1**	**Quintile 2**	**Quintile 3**	**Quintile 4**	**Quintile 5**	*P*-value*
Energy intake (Kcal/day)	1,800.6 ± 613.2^†^	1,807.9 ± 644.3	1,829.1 ± 644.4	1,831.6 ± 655.4	1,848.4 ± 636.3	0.14
Fiber intake (g/day)	12.16 ± 13.50	19.52 ± 20.70	26.56 ± 24.27	33.87 ± 28.52	38.16 ± 27.75	<0.0001
Sodium intake (mg/day)	7,336 ± 4,550	7,384 ± 4,439	7,331 ± 4,848	75,02 ± 5,168	7,285 ± 4,346	0.98
Potassium intake (mg/day)	1,783 ± 1,369	2,447 ± 2,649	2,265 ± 2,089	2,342 ± 1,813	2,636 ± 1,655	<0.0001
Fat intake (% energy)	20.02 ± 9.23	15.14 ± 9.39	14.99 ± 9.59	14.05 ± 9.34	14.87 ± 9.81	<0.0001
Carbohydrate intake (% energy)	64.35 ± 11.83	70.96 ± 11.57	71.02 ± 12.07	71.81 ± 11.90	70.59 ± 12.24	<0.0001
Protein intake (% energy)	
All	13.52 ± 3.29	12.25 ± 2.77	12.97 ± 2.25	14.15 ± 1.93	16.81 ± 2.48	<0.0001
Plant foods	7.91 ± 1.18	9.86 ± 0.34	11.11 ± 0.37	12.71 ± 0.57	15.77 ± 2.09	<0.0001
Animal foods	5.61 ± 3.61	2.39 ± 2.79	1.86 ± 2.24	1.44 ± 1.87	1.04 ± 1.53	<0.0001
Grains	6.03 ± 1.36	7.38 ± 1.49	7.98 ± 1.70	8.98 ± 2.11	9.80 ± 2.78	<0.0001
Tubers	0.20 ± 0.36	0.45 ± 0.87	0.55 ± 0.97	0.47 ± 0.83	0.30 ± 0.56	0.0216
Beans	0.62 ± 0.79	0.92 ± 1.16	1.49 ± 1.40	2.06 ± 1.99	4.63 ± 3.78	<0.0001
Vegetables	0.95 ± 0.63	1.06 ± 0.89	1.01 ± 0.81	1.13 ± 0.96	1.00 ± 0.94	0.11
Fruits	0.03 ± 0.07	0.01 ± 0.04	0.01 ± 0.08	0.00 ± 0.02	0.00 ± 0.03	0
Nuts	0.08 ± 0.38	0.05 ± 0.28	0.08 ± 0.41	0.07 ± 0.40	0.04 ± 0.21	0.13
Red meat	2.85 ± 2.35	1.27 ± 1.73	1.01 ± 1.37	0.82 ± 1.09	0.59 ± 0.91	<0.0001
Poultry	0.39 ± 1.05	0.10 ± 0.43	0.06 ± 0.34	0.05 ± 0.33	0.03 ± 0.30	<0.0001
Fish/shrimp	0.84 ± 1.67	0.29 ± 0.92	0.23 ± 0.93	0.16 ± 0.72	0.07 ± 0.51	<0.0001
Dairy	0.32 ± 0.76	0.14 ± 0.59	0.11 ± 0.47	0.08 ± 0.37	0.06 ± 0.30	<0.0001
Eggs	0.91 ± 1.13	0.51 ± 0.95	0.38 ± 0.66	0.28 ± 0.62	0.25 ± 0.57	<0.0001

^*^ANOVA was used to test the difference of dietary intakes across quintiles of plant protein intake and Chi-square for categorical variables.

^†^All such data were means ± standard deviations.

### Protein intake

Participants consumed 13.94% of the energy intake from total protein, with 11.47% from plant protein and 2.47% from animal protein. The main plant sources of protein were grains 8.03% of total energy intake), beans (1.94%), and vegetables (1.03%). The main animal sources of protein included red meat (1.31%), eggs (0.46%), and fish/shrimp (0.32%).

### Cognitive decline

During a median follow-up of 9 (2–18) years, composite cognitive Z-score declined by 0.4 (−0.4 ± 1.1) SD. The prevalence of cognitive decline defined by the cognitive change below mean minus 1.5 SDs and below mean minus 2 SDs was 7.0 and 2.3%, respectively.

### Protein intake and the change in composite cognitive Z-score

Individuals in quintile 5 of plant protein intake had a higher decrease in composite cognitive Z-score [β (95% confidence interval (CI)): −0.17 (−0.28, −0.06) SD] compared with those in quintile 1. Each 1% increment in energy intake from grain protein was found to represent a 0.03 SD decrease in composite cognitive Z-score. Protein intake from other plant foods was not significantly associated with change in composite cognitive Z-score.

Conversely, animal protein intake was positively associated with change in composite cognitive Z-score. Each 1% increment in energy intake from animal protein was found to represent a 0.01 SD increase in composite cognitive Z-score. The corresponding number for protein intake from poultry and fish was 0.08 SD and 0.03 SD, respectively. Protein intake from red meat, dairy, or eggs was positively associated with change in composite cognitive Z-score before but not after adjustment for the intake of energy, fiber, sodium, potassium, and fat (Table 3).

**Table 3:** Protein intake from different food sources and the change in composite cognitive score^*^.

**Protein intake**	**Consumption level**						**Each 1% energy**
**(% Energy)**	**Quintile 1**	**Quintile 2**	**Quintile 3**	**Quintile 4**	**Quintile 5**	***P*-trend**	
**All foods**	
Range	<11.36	11.36–12.86	12.86–14.27	14.28–16.15	>16.15		
Participants	617	616	616	617	617		
β (95% CI), Model 1^†^	Reference	0.09 (−0.02, 0.19)	−0.02 (−0.12, 0.09)	0.01 (−0.10, 0.11)	0.11 (0.01, 0.21)	0.24	0.01 (0.00, 0.02)
β (95% CI), Model 2^‡^	Reference	0.03 (−0.07, 0.13)	−0.06 (−0.16, 0.04)	−0.09 (−0.19, 0.01)	−0.01 (−0.11, 0.09)	0.23	−0.00 (−0.02, 0.01)
β (95% CI), Model 3^§^	Reference	0.01 (−0.09, 0.12)	−0.06 (−0.17, 0.04)	−0.09 (−0.20, 0.01)	−0.03 (−0.14, 0.07)	0.15	−0.00 (−0.02, 0.01)
**Plant foods**	
Range	<9.26	9.26–10.45	10.46–11.80	11.81–13.71	>13.71		
Participants	617	616	616	617	617		
β (95% CI), Model 1	Reference	−0.13 (−0.24, −0.03)	−0.18 (−0.28, −0.07)	−0.22 (−0.32, −0.11)	−0.29 (−0.40, −0.19)	<0.0001	−0.03 (−0.04, −0.02)
β (95% CI), Model 2	Reference	−0.06 (−0.16, 0.04)	−0.10 (−0.20, 0.01)	−0.12 (−0.23, −0.02)	−0.21 (−0.31, −0.10)	0.0001	−0.02 (−0.03, −0.01)
β (95% CI), Model 3	Reference	−0.03 (−0.13, 0.08)	−0.06 (−0.17, 0.04)	−0.08 (−0.18, 0.03)	−0.17 (−0.28, −0.06)	0.0026	−0.02 (−0.03, −0.00)
**Animal foods**	
Range	0	0–0.88	0.88–2.07	2.08–4.17	>4.17		
Participants	745	488	616	617	617		
β (95% CI), Model 1	Reference	0.00 (−0.10, 0.11)	0.01 (−0.09, 0.11)	0.27 (0.17, 0.37)	0.31 (0.21, 0.41)	<0.0001	0.04 (0.03, 0.05)
β (95% CI), Model 2	Reference	−0.04 (−0.14, 0.07)	−0.03 (−0.13, 0.07)	0.19 (0.09, 0.29)	0.14 (0.03, 0.25)	0.0002	0.02 (0.01, 0.03)
β (95% CI), Model 3	Reference	−0.03 (−0.14, 0.07)	−0.04 (−0.14, 0.06)	0.15 (0.05, 0.26)	0.08 (−0.04, 0.21)	0.0227	0.01 (0.00, 0.03)
**Grains**	
Range	<6.10	6.10–7.26	7.27–8.46	8.47–9.86	>9.87		
Participants	617	616	616	617	617		
β (95% CI), Model 1	Reference	−0.07 (−0.17, 0.03)	−0.06 (−0.16, 0.04)	−0.21 (−0.31, −0.10)	−0.28 (−0.38, −0.18)	<0.0001	−0.04 (−0.05, −0.03)
β (95% CI), Model 2	Reference	0.00 (−0.10, 0.10)	0.02 (−0.08, 0.13)	−0.11 (−0.22, −0.01)	−0.17 (−0.28, −0.07)	0.0002	−0.02 (−0.04, −0.01)
β (95% CI), Model 3	Reference	−0.01 (−0.11, 0.10)	0.02 (−0.09, 0.13)	−0.12 (−0.23, −0.01)	−0.16 (−0.28, −0.05)	0.0008	−0.03 (−0.04, −0.01)
**Tubers**	
Range	0	0–0.01	0.02–0.18	0.19–0.55	>0.55		
Participants	1,208	25	616	617	617		
β (95% CI), Model 1	Reference	0.21 (−0.15, 0.57)	0.03 (−0.06, 0.12)	0.06 (−0.03, 0.15)	0.01 (−0.08, 0.10)	0.51	−0.02 (−0.06, 0.03)
β (95% CI), Model 2	Reference	0.20 (−0.15, 0.55)	0.00 (−0.09, 0.09)	0.02 (−0.07, 0.11)	0.04 (−0.05, 0.13)	0.43	0.02 (−0.02, 0.06)
β (95% CI), Model 3	Reference	0.21 (−0.14, 0.56)	−0.00 (−0.09, 0.09)	0.02 (−0.07, 0.11)	0.09 (0.00, 0.18)	0.11	0.04 (−0.00, 0.09)
**Beans**	
Range	0	0–0.59	0.60–1.64	1.65–3.32	>3.32		
Participants	776	457	616	617	617		
β (95% CI), Model 1	Reference	0.03 (−0.08, 0.14)	−0.02 (−0.12, 0.08)	0.01 (−0.09, 0.11)	−0.00 (−0.10, 0.09)	0.89	−0.00 (−0.02, 0.01)
β (95% CI), Model 2	Reference	0.01 (−0.09, 0.12)	−0.05 (−0.15, 0.04)	−0.02 (−0.12, 0.07)	−0.03 (−0.13, 0.06)	0.39	−0.01 (−0.02, 0.00)
β (95% CI), Model 3	Reference	0.01 (−0.10, 0.11)	−0.06 (−0.16, 0.04)	−0.04 (−0.13, 0.06)	−0.04 (−0.13, 0.06)	0.30	−0.01 (−0.02, 0.01)
**Vegetables**	
Range	<0.40	0.40–0.67	0.68–0.99	1.00–1.51	>1.51		
Participants	617	616	616	617	617		
β (95% CI), Model 1	Reference	−0.02 (−0.12, 0.09)	0.04 (−0.07, 0.14)	0.01 (−0.10, 0.11)	−0.02 (−0.12, 0.09)	0.92	−0.02 (−0.05, 0.02)
β (95% CI), Model 2	Reference	−0.05 (−0.15, 0.05)	0.01 (−0.09, 0.11)	−0.01 (−0.11, 0.10)	−0.02 (−0.12, 0.08)	0.96	−0.01 (−0.05, 0.03)
β (95% CI), Model 3	Reference	−0.05 (−0.15, 0.05)	0.01 (−0.09, 0.12)	0.01 (−0.09, 0.11)	0.03 (−0.08, 0.13)	0.37	0.01 (−0.03, 0.05)
**Fruits**	
Range	0	>0					
Participants	2,869	214					
β (95% CI), Model 1	Reference	0.23 (0.10, 0.36)				0.0004	0.45 (−0.15, 1.04)
β (95% CI), Model 2	Reference	0.07 (−0.06, 0.20)				0.27	−0.19 (−0.78, 0.41)
β (95% CI), Model 3	Reference	0.04 (−0.09, 0.17)				0.54	−0.21 (−0.81, 0.38)
**Nuts**	
Range	0	>0					
Participants	2,854	229					
β (95% CI), Model 1	Reference	0.11 (−0.01, 0.24)				0.0779	0.12 (0.02, 0.21)
β (95% CI), Model 2	Reference	0.03 (−0.10, 0.15)				0.67	0.05 (−0.05, 0.14)
β (95% CI), Model 3	Reference	0.02 (−0.11, 0.14)				0.80	0.04 (−0.06, 0.13)
**Red meat**	
Range	0	0–0.35	0.36–1.08	1.09–2.30	>2.30		
Participants	1,094	139	616	617	617		
β (95% CI), Model 1	Reference	−0.15 (−0.31, 0.01)	0.04 (−0.06, 0.13)	0.12 (0.03, 0.21)	0.27 (0.17, 0.36)	<0.0001	0.04 (0.03, 0.06)
β (95% CI), Model 2	Reference	−0.20 (−0.36, −0.04)	−0.01 (−0.10, 0.08)	0.06 (−0.04, 0.15)	0.13 (0.04, 0.23)	0.0058	0.01 (−0.01, 0.03)
β (95% CI), Model 3	Reference	−0.21 (−0.37, −0.05)	−0.02 (−0.12, 0.07)	0.03 (−0.06, 0.13)	0.08 (−0.03, 0.18)	0.11	0.01 (−0.02, 0.03)
**Poultry**	
Range	0	>0					
Participants	2,850	233					
β (95% CI), Model 1	Reference	0.29 (0.16, 0.41)				<0.0001	0.13 (0.08, 0.19)
β (95% CI), Model 2	Reference	0.18 (0.06, 0.30)				<0.0001	0.08 (0.03, 0.14)
β (95% CI), Model 3	Reference	0.15 (0.03, 0.27)				0.0180	0.08 (0.02, 0.13)
**Fish/shrimp**	
Range	0	>0					
Participants	2,630	453					
β (95% CI), Model 1	Reference	0.32 (0.22, 0.41)				<0.0001	0.08 (0.05, 0.11)
β (95% CI), Model 2	Reference	0.19 (0.09, 0.28)				0.0001	0.04 (0.01, 0.07)
β (95% CI), Model 3	Reference	0.16 (0.06, 0.26)				0.0012	0.03 (0.00, 0.06)
**Dairy**	
Range	0	>0					
Participants	2,757	326					
β (95% CI), Model 1	Reference	0.13 (0.03, 0.24)				0.0145	0.04 (−0.02, 0.10)
β (95% CI), Model 2	Reference	0.02 (−0.09, 0.12)				0.73	−0.01 (−0.07, 0.05)
β (95% CI), Model 3	Reference	0.00 (−0.10, 0.11)				0.94	−0.01 (−0.07, 0.05)
**Eggs**	
Range	<0.17	0.18–0.86	>0.86				
Participants	1,849	617	617				
β (95% CI), Model 1	Reference	0.10 (0.01, 0.18)	0.19 (0.10, 0.27)			<0.0001	0.09 (0.05, 0.13)
β (95% CI), Model 2	Reference	0.03 (−0.06, 0.11)	0.06 (−0.03, 0.15)			0.17	0.04 (−0.01, 0.08)
β (95% CI), Model 3	Reference	0.01 (−0.08, 0.09)	0.02 (−0.08, 0.11)			0.72	0.03 (−0.01, 0.07)

^*^Generalized linear regression models were used to obtain coefficients for the change in composite cognitive score for quintiles 2–5 vs. the quintile 1 and per 1% increment in energy intake from protein intake. We used the Benjamin–Hochberg procedure to control the false discovery rate at level 5% for multiple comparisons with the P-value cut-off point of significance was 0.0179. The change in composite cognitive score was computed as the score at baseline subtracting from that at follow-up.

^†^Model 1 was adjusted for age and gender.

^‡^Model 2 was adjusted for Model 1 plus education, urbanization, years of follow-up, smoking, alcohol intake, physical activity, composite cognitive score, diabetes, BMI, systolic, and diastolic blood pressure at baseline.

^§^Model 3 was adjusted for model 2 plus intake of energy, sodium, potassium, fat, and fiber.

### Protein intake and changes in Z-scores of memory, subtraction, and backward counting tests

Plant protein intake was inversely associated with change in memory Z-score [β (95% CI) for quintile 5 vs. quintile 1: −0.79 (−1.32, −0.26)] in the multivariable analysis. Animal protein intake was positively associated with change in memory Z-score before but not after adjustment for confounders (Supplementary Table 2). An inverse association between plant protein intake and change in subtraction Z-score was found before but not after adjustment for confounders. Animal protein intake was positively associated with change in subtraction Z-score [β (95% CI) for quintile 5 vs. quintile 1: 0.34 (0.09, 0.58)] (Supplementary Table 3). In the multivariable analysis, plant protein intake was inversely [β (95% CI) for quintile 5 vs. quintile 1: −0.08 (−0.15, −0.01)] but animal protein intake was positively [0.09 (0.01, 0.17)] associated with change in backward counting Z-score (Supplementary Table 4).

### Protein intake and cognitive decline

Participants in quintile 5 of plant protein intake had a higher risk [odds ratio (OR) (95% CI): 3.03 (1.22–7.53)] of cognitive decline as defined by cognitive change below mean minus 2 SDs compared with those in quintile 1 (Table 4). This was consistent with cognitive decline as defined by cognitive change below mean minus 1.5 SDs [OR (95% CI) for quintile 5 vs. quintile 1 of plant protein intake: 1.62 (0.99–2.67)]. Animal protein intake was not significantly associated with cognitive decline (Supplementary Table 5).

**Table 4:** Protein intake from different sources and cognitive decline^*^.

**Protein intake**	**Consumption level**					
**(% Energy)**	**Quintile 1**	**Quintile 2**	**Quintile 3**	**Quintile 4**	**Quintile 5**	***P*-trend**
**All**	
Range	<11.36	11.36–12.86	12.86–14.27	14.28–16.15	>16.15	
Events/Participants	12/617	9/616	19/616	12/617	18/617	
OR (95% CI), Model 1^†^	1.00	0.66 (0.27–1.63)	1.59 (0.77–3.31)	0.99 (0.44–2.23)	1.42 (0.67–3.01)	0.23
OR (95% CI), Model 2^‡^	1.00	0.56 (0.22–1.45)	1.60 (0.76–3.35)	1.02 (0.45–2.33)	1.44 (0.67–3.13)	0.17
OR (95% CI), Model 3^§^	1.00	0.56 (0.22–1.47)	1.51 (0.70–3.27)	1.03 (0.44–2.41)	1.43 (0.64–3.20)	0.18
**Plant foods**	
Range	<9.26	9.26–10.45	10.46–11.80	11.81–13.71	>13.71	
Events/Participants	9/617	16/616	11/616	14/617	20/617	
OR (95% CI), Model 1	1.00	1.71 (0.74–3.94)	1.24 (0.51–3.02)	1.59 (0.68–3.70)	2.17 (0.97–4.85)	0.0966
OR (95% CI), Model 2	1.00	2.14 (0.88–5.17)	1.55 (0.61–3.96)	1.95 (0.79–4.82)	2.82 (1.19–6.72)	0.0429
OR (95% CI), Model 3	1.00	2.22 (0.91–5.44)	1.56 (0.60–4.06)	2.09 (0.82–5.32)	3.03 (1.22–7.53)	0.0373
**Animal foods**	
Range	0	0–0.88	0.88–2.07	2.08–4.17	>4.17	
Events/Participants	20/745	10/488	16/616	12/617	12/617	
OR (95% CI), Model 1	1.00	0.81 (0.37–1.75)	0.95 (0.48–1.89)	0.75 (0.36–1.56)	0.75 (0.36–1.55)	0.41
OR (95% CI), Model 2	1.00	0.79 (0.36–1.73)	0.86 (0.42–1.75)	0.64 (0.29–1.40)	0.57 (0.24–1.37)	0.18
OR (95% CI), Model 3	1.00	0.73 (0.33–1.62)	0.74 (0.36–1.54)	0.55 (0.24–1.26)	0.49 (0.19–1.26)	0.11

^*^Logistic regression models were used to estimate ORs (95% CIs) for cognitive decline associated with protein intake from different sources. Cognitive decline was defined as change in the composite cognitive score below the mean minus 2 SDs.

^†^Model 1 was adjusted for age and gender.

^‡^Model 2 was adjusted for Model 1 plus education, urbanization, years of follow-up, smoking, alcohol intake, physical activity, composite cognitive score, diabetes, BMI, systolic, and diastolic blood pressure at baseline.

^§^Model 3 was adjusted for model 2 plus intake of energy, sodium, potassium, fat, and fiber.

### Composition of protein sources and the change in composite cognitive Z-score

Compared with participants in quintile 1 of animal protein (% total protein), those in the highest quintile had a higher increase in composite cognitive Z-score [0.14 (95% CI: 0.02, 0.27) SD)]. High animal protein intake was associated with a lower risk of cognitive decline defined by cognitive change below mean minus 2 SDs [OR (95% CI) for quintile 5 vs. quintile 1: 0.22 (0.07–0.71)] (Table 5).

**Table 5:** Composition of animal and plant protein sources and cognitive decline^*^.

	**Animal protein intake (% total protein)**					
	**Quintile 1**	**Quintile 2**	**Quintile 3**	**Quintile 4**	**Quintile 5**	***P*-trend**
Range	0	0–6.63	6.64–15.70	15.71–30.71	>30.71	
Participants	745	488	616	617	617	
**Change in composite cognitive score**	
β (95% CI), Model 1^†^	0	0.00 (−0.10, 0.11)	0.03 (−0.06, 0.13)	0.23 (0.13, 0.33)	0.35 (0.24, 0.45)	<0.0001
β (95% CI), Model 2^‡^	0	−0.03 (−0.14, 0.07)	−0.00 (−0.10, 0.10)	0.16 (0.05, 0.26)	0.20 (0.09, 0.32)	<0.0001
β (95% CI), Model 3^§^	0	−0.03 (−0.13, 0.08)	−0.02 (−0.12, 0.08)	0.12 (0.01, 0.22)	0.14 (0.02, 0.27)	0.0083
**Cognitive decline** ^**¶**^	
Events	20	12	13	15	10	
OR (95% CI), Model 1	1.00	1.01 (0.45–2.28)	0.60 (0.27–1.33)	0.44 (0.20–0.95)	0.29 (0.12–0.66)	0.0007
OR (95% CI), Model 2	1.00	1.07 (0.46–2.47)	0.63 (0.27–1.44)	0.43 (0.18–1.05)	0.24 (0.08–0.70)	0.0034
OR (95% CI), Model 3	1.00	0.91 (0.38–2.20)	0.53 (0.21–1.28)	0.34 (0.13–0.90)	0.22 (0.07–0.71)	0.0042
**Cognitive decline** ^**?**^	
Events	55	28	42	52	39	
OR (95% CI), Model 1	1.00	0.70 (0.41–1.18)	0.72 (0.45–1.16)	0.63 (0.40–1.00)	0.38 (0.24–0.62)	0.0002
OR (95% CI), Model 2	1.00	0.74 (0.43–1.28)	0.79 (0.48–1.31)	0.76 (0.46–1.24)	0.46 (0.26–0.83)	0.0278
OR (95% CI), Model 3	1.00	0.74 (0.42–1.30)	0.82 (0.49–1.37)	0.88 (0.52–1.51)	0.56 (0.29–1.07)	0.21

^*^Generalized linear regression models were used to obtain coefficients for the change in composite cognitive score for quintiles 2–5 vs. the quintile 1 of the composition of animal and plant protein intake. Logistic regression models were used to estimate ORs (95% CIs) for cognitive decline associated with the composition of animal and plant protein intake.

^†^Model 1 was adjusted for age and gender.

^‡^Model 2 was adjusted for Model 1 plus education, urbanization, years of follow-up, smoking, alcohol intake, physical activity, composite cognitive score, diabetes, BMI, systolic and diastolic blood pressure at baseline.

^§^Model 3 was adjusted for model 2 plus intake of energy, sodium, potassium, fat, and fiber.

^¶^Cognitive decline was defined as the change in the composite cognitive score below the mean minus two standard deviations.

^?^Cognitive decline was defined as the change in the composite cognitive score below the mean minus 1.5 standard deviations.

### Moderation analysis

No significant interaction (all *P*-values for interaction >0.05) between animal/plant protein intake and important factors examined with the change in composite cognitive Z-score was observed (data not shown).

### Sensitivity analysis

Sensitivity analysis showed the average annual plant protein intake reported in surveys completed before the first cognitive assessment was inversely associated with change in composite cognitive Z-score during follow-up (Supplementary Table 6).

## Discussion

This longitudinal study of community-dwelling older Chinese adults demonstrated that lower plant protein but higher animal protein intake was associated with a lower risk of cognitive decline. Lower protein intake from grains and higher protein intake from poultry and fish/shrimp, were associated with a lower rate of cognitive decline.

We found higher plant protein intake was associated with greater cognitive decline. Our findings are supported by some studies from Europe demonstrating a positive association between plant protein intake and diabetes (25). The harmful effect of high plant protein intake may be attributed to the deficiency of micronutrients in plant-based diets including vitamin B12 and iron, which are associated with a higher risk of cognitive impairment (26–28). Furthermore, plant-based proteins have relatively low essential amino acids and leucine contents or even lack one or more of the essential amino acids when compared with animal-based proteins, such that, a higher plant protein intake is less likely to increase lean and skeletal muscle mass (29). This may explain the inverse association between plant protein intake was associated and change in cognition. Higher protein intake from grains was independently associated with accelerated cognitive decline. This may be due to the fact that grains contain relatively low quantities of essential amino acid lysine, of which lower intake may increase the risk of hypertension, diabetes and obesity (30, 31) resulting in cognitive decline.

We found an inverse association between animal protein intake and cognitive decline. A meta-analysis showing that higher animal protein intake was associated with a lower risk of stroke [RR (95% CI): 0.71 (0.50–0.99)] is consistent with our findings (32). Our findings are also consistent with some studies of Japanese and Chinese populations demonstrating that animal protein intake was inversely associated with blood pressure (33, 34). Animal proteins usually contain all essential amino acids, and therefore may consist of optimal amino acid composition resulting in better metabolic health (29). A recent longitudinal study demonstrates that an adequate methionine (mainly from animal foods) status may decrease the risk of dementia and brain atrophy (35). We observed higher fish protein intake was independently associated with a lower risk of cognitive decline, which is consistent with a study showing that ≥1 servings/week of fish intake was associated with a reduced cognitive decline rate in adults aged ≥ 65 years (36). We also found an inverse association between poultry protein intake and the risk of cognitive decline. Our findings are supported by a prospective study demonstrating that higher poultry intake was associated with less cognitive decline over 6 years in older Swedish adults (7). Protein intake from red meat, dairy, or eggs was not independently associated with cognitive decline suggesting potential beneficial effects of higher animal protein intake were driven by fish/shrimp and poultry.

An optimal amino acid composition of dietary protein intake may help optimize amino acid metabolism and protect against dementia risks including obesity, diabetes, hypertension, and stroke (37). A cross-sectional study of 661 Chinese adults found that a higher total protein intake was associated with a higher likelihood of mild cognitive impairment (38). Likely, we found higher total protein intake was associated with accelerated cognitive decline. Notably, animal sources accounted for only 16.3% of total protein intake in our study, which was much lower than that reported in individuals from the USA, Europe, and Australia (64–75%) as well as in Japan (54%) (25, 39–42). Meanwhile, plant protein intake was inversely but animal protein intake was positively associated with change in composite cognitive Z-score suggesting the inverse association between total protein intake and cognition was driven by plant protein in our study. This indicates that increasing the proportion of animal protein in populations with plant dominant diets may help protect against cognitive decline. A recent study of US women and men demonstrated that higher plant protein intake and lower animal protein intake was associated with lower likelihood of cognitive decline (13), but other studies showed that specific protein food sources were not significantly associated with cognitive function (14).

The conflicting findings between our study and Yeh et al. may be due to the fact that the plant foods are dominant in our population, but animal foods are dominant in the US population.

To our knowledge, this is the first longitudinal study to examine the association between protein intakes from different food sources with cognitive decline. Our study has several limitations. First, cognitive assessment was conducted in a subgroup of the CHNS cohort, which limits the generalization of our findings to the whole population in China. Second, our study was conducted in a population with plant food dominant diets, therefore, more longitudinal studies in populations with animal food dominant diets are needed to warrant our findings. Finally, the wide range of follow-up in our study might influence associations between protein intake and cognition. However, the results did not substantially change after adjusting for follow-up and the follow-up did not mediate the association, suggesting our findings are independent of the follow-up duration.

In conclusion, the intake of protein from plant foods especially grains were positively associated but the intake of protein from animal foods, especially fish/shrimp and poultry, were inversely associated with accelerated cognitive decline. A relatively high proportion of animal protein in population with plant dominant diets may be protective of cognitive decline.

## Data availability statement

The original contributions presented in the study are included in the article/Supplementary material, further inquiries can be directed to the corresponding author/s.

## Author contributions

RG, ZY, and WY conceived and designed the study. RG, WD, and YZ conducted data analysis and drafted the initial manuscript. ZY, WY, and FZ made critical revision of the manuscript for important intellectual content. All authors read the manuscript and approved the final draft.

## Funding

This research uses data from China Health and Nutrition Survey (CHNS). We thank the National Institute for Nutrition and Health, China Center for Disease Control and Prevention, Carolina Population Center (P2C HD050924 and T32 HD007168), the University of North Carolina at Chapel Hill, the NIH (R01-HD30880, DK056350, R24 HD050924, and R01-HD38700) and the NIH Fogarty International Center (D43 TW009077 and D43 TW007709) for financial support for the CHNS data collection and analysis files from 1989 to 2015 and future surveys, and the China-Japan Friendship Hospital, Ministry of Health for support for CHNS 2009, Chinese National Human Genome Center at Shanghai since 2009, and Beijing Municipal Center for Disease Prevention and Control since 2011.

## Conflict of interest

The authors declare that the research was conducted in the absence of any commercial or financial relationships that could be construed as a potential conflict of interest.

## Publisher's note

All claims expressed in this article are solely those of the authors and do not necessarily represent those of their affiliated organizations, or those of the publisher, the editors and the reviewers. Any product that may be evaluated in this article, or claim that may be made by its manufacturer, is not guaranteed or endorsed by the publisher.

## Abbreviations

BMI: body mass index
CHNS: china health and nutrition survey
CI: confidence interval
MET: metabolic equivalent of task
OR: odds ratio
SD: standard deviation.
