# Supplementary material for: Protein intake from different sources and cognitive decline over 9 years in community-dwelling older adults
Source: Front Public Health. 2022 Oct 14;10:1016016. doi: 10.3389/fpubh.2022.1016016 (PMC9614310; doi:10.3389/fpubh.2022.1016016)
Supplement: Supplementary file 1 [file Table_1.DOCX]

**Table S1. Baseline characteristics across quintiles of animal protein intake**

**Table S2. Protein intake from different food sources and the change in memory Z-score**

**Table S3. Protein intake from different food sources and the change in subtraction Z-score**

**Table S4. Protein intake from different food sources and the change in backward counting Z-score**

**Table S5. Protein intake from different sources and cognitive decline**

**Table S6. The average animal protein intake from different food sources of surveys until the first cognitive assessment and the change in composite cognitive score**

**Table S1. Baseline characteristics across quintiles of animal protein intake**

|  | Animal protein intake | | | | |  |  |
| --- | --- | --- | --- | --- | --- | --- | --- |
|  | Quintile 1 | Quintile 2 | Quintile 3 | Quintile 4 | Quintile 5 |  | P-value* |
| Age (years) | 60.84 ± 6.19^†^ | 60.61 ± 6.00 | 62.31 ± 6.61 | 62.39 ± 6.69 | 63.06 ± 6.91 |  | <0.0001 |
| Gender |  |  |  |  |  |  | 0.89 |
| Men | 353 (47.4)^‡^ | 236 (48.4) | 307 (49.8) | 310 (50.2) | 288 (46.7) |  |  |
| Women | 392 (52.6) | 252 (51.6) | 309 (50.2) | 307 (49.8) | 329 (53.3) |  |  |
| Education |  |  |  |  |  |  | <0.0001 |
| Illiterate | 443 (59.5) | 223 (45.7) | 299 (48.5) | 241 (39.1) | 152 (24.6) |  |  |
| Primary school | 203 (27.2) | 138 (28.3) | 163 (26.5) | 162 (26.3) | 120 (19.4) |  |  |
| Lower middle school | 60 (8.1) | 75 (15.4) | 83 (13.5) | 86 (13.9) | 121 (19.6) |  |  |
| Upper middle school | 8 (1.1) | 13 (2.7) | 20 (3.2) | 39 (6.3) | 83 (13.5) |  |  |
| Technical or vocational | 9 (1.2) | 8 (1.6) | 20 (3.2) | 43 (7.0) | 69 (11.2) |  |  |
| University or college |  | 7 (1.4) | 11 (1.8) | 24 (3.9) | 42 (6.8) |  |  |
| Missing | 22 (3.0) | 24 (4.9) | 20 (3.2) | 22 (3.6) | 30 (4.9) |  |  |
| Living area |  |  |  |  |  |  | <0.0001 |
| Urban | 83 (11.1) | 111 (22.7) | 215 (34.9) | 311 (50.4) | 419 (67.9) |  |  |
| Rural | 662 (88.9) | 377 (77.3) | 401 (65.1) | 306 (49.6) | 198 (32.1) |  |  |
| Smoking |  |  |  |  |  |  | 0.0079 |
| Never | 491 (65.9) | 326 (66.8) | 387 (62.8) | 407 (66.0) | 456 (73.9) |  |  |
| Former | 26 (3.5) | 11 (2.3) | 21 (3.4) | 18 (2.9) | 25 (4.1) |  |  |
| Current | 228 (30.6) | 151 (30.9) | 208 (33.8) | 192 (31.1) | 136 (22.0) |  |  |
| Alcohol intake |  |  |  |  |  |  | 0.71 |
| None | 501 (67.2) | 334 (68.4) | 421 (68.3) | 408 (66.1) | 428 (69.4) |  |  |
| Yes | 244 (32.8) | 154 (31.6) | 195 (31.7) | 209 (33.9) | 189 (30.6) |  |  |
| Diabetes |  |  |  |  |  |  | <0.0001 |
| No | 735 (98.7) | 483 (99.0) | 601 (97.6) | 588 (95.3) | 583 (94.5) |  |  |
| Yes | 10 (1.3) | 5 (1.0) | 15 (2.4) | 29 (4.7) | 34 (5.5) |  |  |
| Composite cognitive score (Z-score) | -0.08 ± 0.91 | -0.05 ± 0.87 | 0.11 ± 0.87 | 0.21 ± 0.92 | 0.34 ± 0.86 |  | <0.0001 |
| Memory Z-score | 9.37 ± 4.33 | 9.31 ± 4.18 | 9.74 ± 4.33 | 10.18 ± 4.63 | 10.64 ± 4.34 |  | <0.0001 |
| Subtraction Z-score | 3.25 ± 1.97 | 3.52 ± 1.87 | 3.68 ± 1.79 | 3.76 ± 1.75 | 3.96 ± 1.63 |  | <0.0001 |
| Backward counting Z-score | 0.99 ± 0.56 | 1.00 ± 0.48 | 1.02 ± 0.48 | 1.03 ± 0.42 | 1.01 ± 0.40 |  | 0.37 |
| Physical activity (MET-h/wk) | 243.8 ± 114.3 | 226.1 ± 114.1 | 198.1 ± 114.2 | 155.9 ± 108.3 | 118.4 ± 94.6 |  | <0.0001 |
| BMI (kg/m^2^) | 22.23 ± 3.34 | 22.70 ± 3.64 | 23.00 ± 3.49 | 23.52 ± 3.60 | 24.04 ± 3.46 |  | <0.0001 |
| Diastolic blood pressure (mmHg) | 80.87 ± 12.35 | 80.30 ± 11.81 | 80.60 ± 11.15 | 81.75 ± 11.87 | 82.46 ± 10.50 |  | 0.0025 |
| Systolic blood pressure (mmHg) | 127.63 ± 20.72 | 127.74 ± 19.95 | 129.56 ± 20.26 | 129.85 ± 19.78 | 130.91 ± 17.51 |  | 0.0005 |
| Energy intake (Kcal/day) | 1898.1 ± 706.4 | 1858.3 ± 644.3 | 1826.4 ± 654.0 | 1787.9 ± 578.4 | 1738.7 ± 576.4 |  | <0.0001 |
| Fiber intake (g/day) | 30.29 ± 28.94 | 36.72 ± 30.14 | 30.12 ± 25.16 | 23.18 ± 20.27 | 11.33 ± 9.95 |  | <0.0001 |
| Sodium intake (mg/day) | 7045 ± 4525 | 7489 ± 5241 | 7339 ± 4740 | 7413 ± 4247 | 7644 ± 4726 |  | 0.0365 |
| Potassium intake (mg/day) | 2537 ± 2152 | 2926 ± 2822 | 2312 ± 1835 | 1994 ± 1412 | 1785 ± 1297 |  | <0.0001 |
| Fat intake (% energy) | 9.96 ± 8.02 | 13.48 ± 8.52 | 15.99 ± 9.57 | 18.80 ± 9.14 | 21.51 ± 8.61 |  | <0.0001 |
| Carbohydrate intake (% energy) | 77.22 ± 10.20 | 72.54 ± 11.45 | 69.75 ± 11.82 | 66.30 ± 11.06 | 62.02 ± 10.53 |  | <0.0001 |
| Protein intake (% energy) |  |  |  |  |  |  |  |
| All | 12.66 ± 2.67 | 13.37 ± 2.96 | 13.42 ± 2.31 | 13.98 ± 2.42 | 16.42 ± 3.21 |  | <0.0001 |
| Plant foods | 12.66 ± 2.67 | 12.85 ± 2.94 | 11.98 ± 2.33 | 10.96 ± 2.43 | 8.96 ± 2.23 |  | <0.0001 |
| Animal foods | 0 | 0.52 ± 0.21 | 1.44 ± 0.35 | 3.02 ± 0.61 | 7.46 ± 2.79 |  | <0.0001 |
| Grains | 9.29 ± 2.40 | 8.79 ± 2.16 | 8.59 ± 1.95 | 7.53 ± 1.76 | 5.87 ± 1.57 |  | <0.0001 |
| Tubers | 0.68 ± 1.11 | 0.53 ± 0.87 | 0.35 ± 0.63 | 0.22 ± 0.40 | 0.15 ± 0.23 |  | <0.0001 |
| Beans | 1.65 ± 2.42 | 2.39 ± 3.26 | 1.98 ± 2.43 | 2.11 ± 2.47 | 1.74 ± 2.12 |  | 0.76 |
| Vegetables | 1.00 ± 0.95 | 1.11 ± 0.98 | 1.02 ± 0.79 | 1.01 ± 0.81 | 1.03 ± 0.72 |  | 0.94 |
| Fruits | 0 | 0.00 ± 0.02 | 0.01 ± 0.08 | 0.01 ± 0.03 | 0.03 ± 0.08 |  | <0.0001 |
| Nuts | 0.03 ± 0.34 | 0.03 ± 0.15 | 0.04 ± 0.20 | 0.08 ± 0.40 | 0.13 ± 0.48 |  | <0.0001 |
| Red meat | 0 | 0.35 ± 0.28 | 0.97 ± 0.58 | 1.76 ± 1.05 | 3.53 ± 2.46 |  | <0.0001 |
| Poultry | 0 | 0.00 ± 0.02 | 0.01 ± 0.10 | 0.08 ± 0.36 | 0.55 ± 1.15 |  | <0.0001 |
| Fish/shrimp | 0 | 0.01 ± 0.06 | 0.03 ± 0.20 | 0.20 ± 0.57 | 1.36 ± 1.96 |  | <0.0001 |
| Dairy | 0 | 0.01 ± 0.06 | 0.06 ± 0.23 | 0.23 ± 0.61 | 0.41 ± 0.93 |  | <0.0001 |
| Eggs | 0 | 0.14 ± 0.22 | 0.33 ± 0.48 | 0.65 ± 0.78 | 1.24 ± 1.32 |  | <0.0001 |

^*^ANOVA was used to test the difference of continuous variables across meal patterns and Chi-square for categorical variables.

^†^All such data were means ± standard deviations.

^‡^All such data were frequency (percentage).

**Table S2. Protein intake from different food sources and the change in memory Z-score***

|  | Consumption level | | | | |  |
| --- | --- | --- | --- | --- | --- | --- |
| Protein intake from (% energy) | Quintile 1 | Quintile 2 | Quintile 3 | Quintile 4 | Quintile 5 | P-trend |
| All |  |  |  |  |  |  |
| Range | <11.36 | 11.36-12.86 | 12.86-14.27 | 14.28-16.15 | >16.15 |  |
| Participants | 617 | 616 | 616 | 617 | 617 |  |
| β (95% CI), Model 1 ^‡^ | Reference | 0.20 (-0.29, 0.70) | -0.27 (-0.77, 0.22) | -0.26 (-0.76, 0.23) | 0.24 (-0.26, 0.73) | 0.99 |
| β (95% CI), Model 2 ^§^ | Reference | -0.06 (-0.55, 0.43) | -0.48 (-0.97, 0.01) | -0.69 (-1.18, -0.20) | -0.30 (-0.80, 0.20) | 0.0288 |
| β (95% CI), Model 3 ^¶^ | Reference | -0.06 (-0.61, 0.49) | -0.38 (-1.01, 0.26) | -0.53 (-1.26, 0.20) | -0.23 (-1.04, 0.57) | 0.44 |
| Plant foods |  |  |  |  |  |  |
| Range | <9.26 | 9.26-10.45 | 10.46-11.80 | 11.81-13.71 | >13.71 |  |
| Participants | 617 | 616 | 616 | 617 | 617 |  |
| β (95% CI), Model 1 | Reference | -0.47 (-0.96, 0.02) | -0.71 (-1.20, -0.22) | -1.00 (-1.50, -0.50) | -1.35 (-1.84, -0.86) | <0.0001 |
| β (95% CI), Model 2 | Reference | -0.16 (-0.66, 0.33) | -0.38 (-0.87, 0.11) | -0.62 (-1.13, -0.12) | -1.01 (-1.51, -0.50) | <0.0001 |
| β (95% CI), Model 3 | Reference | -0.04 (-0.54, 0.46) | -0.22 (-0.72, 0.28) | -0.39 (-0.91, 0.13) | -0.79 (-1.32, -0.26) | 0.0014 |
| Animal foods |  |  |  |  |  |  |
| Range | 0 | 0-0.88 | 0.88-2.07 | 2.08-4.17 | >4.17 |  |
| Participants | 745 | 488 | 616 | 617 | 617 |  |
| β (95% CI), Model 1 | Reference | -0.26 (-0.76, 0.24) | -0.14 (-0.61, 0.33) | 0.96 (0.49, 1.43) | 1.11 (0.64, 1.58) | <0.0001 |
| β (95% CI), Model 2 | Reference | -0.43 (-0.93, 0.06) | -0.30 (-0.78, 0.17) | 0.61 (0.12, 1.10) | 0.38 (-0.16, 0.91) | 0.0108 |
| β (95% CI), Model 3 | Reference | -0.38 (-0.88, 0.12) | -0.33 (-0.81, 0.15) | 0.44 (-0.07, 0.95) | 0.09 (-0.49, 0.67) | 0.23 |
| Grains |  |  |  |  |  |  |
| Range | <6.10 | 6.10-7.26 | 7.27-8.46 | 8.47-9.86 | >9.87 |  |
| Participants | 617 | 616 | 616 | 617 | 617 |  |
| β (95% CI), Model 1 | Reference | -0.29 (-0.79, 0.20) | -0.21 (-0.70, 0.29) | -0.69 (-1.18, -0.20) | -1.15 (-1.64, -0.65) | <0.0001 |
| β (95% CI), Model 2 | Reference | 0.02 (-0.47, 0.51) | 0.16 (-0.33, 0.66) | -0.29 (-0.79, 0.21) | -0.70 (-1.22, -0.19) | 0.0034 |
| β (95% CI), Model 3 | Reference | -0.01 (-0.51, 0.49) | 0.15 (-0.36, 0.67) | -0.29 (-0.81, 0.24) | -0.61 (-1.17, -0.05) | 0.0169 |
| Tubers |  |  |  |  |  |  |
| Range | 0 | 0-0.01 | 0.02-0.18 | 0.19-0.55 | >0.55 |  |
| Participants | 1208 | 25 | 616 | 617 | 617 |  |
| β (95% CI), Model 1 | Reference | 1.03 (-0.68, 2.75) | 0.15 (-0.28, 0.58) | 0.19 (-0.24, 0.63) | 0.02 (-0.41, 0.45) | 0.67 |
| β (95% CI), Model 2 | Reference | 0.97 (-0.71, 2.65) | 0.03 (-0.39, 0.45) | 0.03 (-0.40, 0.45) | 0.16 (-0.27, 0.58) | 0.58 |
| β (95% CI), Model 3 | Reference | 1.05 (-0.62, 2.73) | 0.04 (-0.38, 0.46) | 0.03 (-0.39, 0.46) | 0.40 (-0.04, 0.83) | 0.18 |
| Beans |  |  |  |  |  |  |
| Range | 0 | 0-0.59 | 0.60-1.64 | 1.65-3.32 | >3.32 |  |
| Participants | 776 | 457 | 616 | 617 | 617 |  |
| β (95% CI), Model 1 | Reference | 0.02 (-0.49, 0.53) | -0.28 (-0.75, 0.19) | -0.12 (-0.59, 0.35) | -0.31 (-0.78, 0.16) | 0.18 |
| β (95% CI), Model 2 | Reference | -0.06 (-0.56, 0.44) | -0.43 (-0.90, 0.03) | -0.27 (-0.73, 0.19) | -0.43 (-0.89, 0.03) | 0.0478 |
| β (95% CI), Model 3 | Reference | -0.06 (-0.56, 0.44) | -0.46 (-0.92, 0.01) | -0.33 (-0.79, 0.13) | -0.43 (-0.89, 0.03) | 0.0368 |
| Vegetables |  |  |  |  |  |  |
| Range | <0.40 | 0.40-0.67 | 0.68-0.99 | 1.00-1.51 | >1.51 |  |
| Participants | 617 | 616 | 616 | 617 | 617 |  |
| β (95% CI), Model 1 | Reference | -0.30 (-0.80, 0.20) | -0.06 (-0.56, 0.43) | -0.07 (-0.56, 0.43) | -0.28 (-0.77, 0.22) | 0.56 |
| β (95% CI), Model 2 | Reference | -0.42 (-0.91, 0.07) | -0.15 (-0.64, 0.33) | -0.11 (-0.59, 0.38) | -0.26 (-0.74, 0.22) | 0.70 |
| β (95% CI), Model 3 | Reference | -0.41 (-0.90, 0.08) | -0.13 (-0.61, 0.36) | -0.04 (-0.52, 0.45) | -0.06 (-0.57, 0.45) | 0.68 |
| Fruits |  |  |  |  |  |  |
| Range | 0 | >0 |  |  |  |  |
| Participants | 2869 | 214 | . | . | . |  |
| β (95% CI), Model 1 | Reference | 0.85 (0.24, 1.45) |  |  |  | 0.0062 |
| β (95% CI), Model 2 | Reference | 0.15 (-0.47, 0.77) |  |  |  | 0.64 |
| β (95% CI), Model 3 | Reference | -0.03 (-0.67, 0.60) |  |  |  | 0.92 |
| Nuts |  |  |  |  |  |  |
| Range | 0 | >0 |  |  |  |  |
| Participants | 2854 | 229 | . | . | . |  |
| β (95% CI), Model 1 | Reference | 0.42 (-0.17, 1.02) |  |  |  | 0.16 |
| β (95% CI), Model 2 | Reference | 0.06 (-0.53, 0.64) |  |  |  | 0.85 |
| β (95% CI), Model 3 | Reference | 0.03 (-0.56, 0.61) |  |  |  | 0.93 |
| Red meat |  |  |  |  |  |  |
| Range | 0 | 0-0.35 | 0.36-1.08 | 1.09-2.30 | >2.30 |  |
| Participants | 1094 | 139 | 616 | 617 | 617 |  |
| β (95% CI), Model 1 | Reference | -0.86 (-1.64, -0.09) | -0.08 (-0.51, 0.36) | 0.33 (-0.10, 0.77) | 0.96 (0.52, 1.40) | <0.0001 |
| β (95% CI), Model 2 | Reference | -1.08 (-1.85, -0.31) | -0.28 (-0.72, 0.15) | 0.03 (-0.41, 0.48) | 0.38 (-0.10, 0.85) | 0.15 |
| β (95% CI), Model 3 | Reference | -1.05 (-1.82, -0.28) | -0.30 (-0.74, 0.14) | -0.05 (-0.51, 0.41) | 0.15 (-0.35, 0.65) | 0.57 |
| Poultry |  |  |  |  |  |  |
| Range | 0 | >0 |  |  |  |  |
| Participants | 2850 | 233 |  |  |  |  |
| β (95% CI), Model 1 | Reference | 1.01 (0.43, 1.60) |  |  |  | 0.0007 |
| β (95% CI), Model 2 | Reference | 0.55 (-0.04, 1.13) |  |  |  | 0.0686 |
| β (95% CI), Model 3 | Reference | 0.36 (-0.24, 0.95) |  |  |  | 0.24 |
| Fish/shrimp |  |  |  |  |  |  |
| Range | 0 | >0 |  |  |  |  |
| Participants | 2630 | 453 |  |  |  |  |
| β (95% CI), Model 1 | Reference | 1.20 (0.76, 1.65) |  |  |  | <0.0001 |
| β (95% CI), Model 2 | Reference | 0.63 (0.18, 1.09) |  |  |  | 0.0067 |
| β (95% CI), Model 3 | Reference | 0.47 (-0.00, 0.95) |  |  |  | 0.0511 |
| Dairy |  |  |  |  |  |  |
| Range | 0 | >0 |  |  |  |  |
| Participants | 2757 | 326 |  |  |  |  |
| β (95% CI), Model 1 | Reference | 0.41 (-0.10, 0.91) |  |  |  | 0.11 |
| β (95% CI), Model 2 | Reference | -0.08 (-0.59, 0.42) |  |  |  | 0.75 |
| β (95% CI), Model 3 | Reference | -0.11 (-0.62, 0.40) |  |  |  | 0.67 |
| Eggs |  |  |  |  |  |  |
| Range | <0.17 | 0.18-0.86 | >0.86 |  |  |  |
| Participants | 1849 | 617 | 617 |  |  |  |
| β (95% CI), Model 1 | Reference | 0.19 (-0.22, 0.59) | 0.86 (0.45, 1.27) |  |  | <0.0001 |
| β (95% CI), Model 2 | Reference | -0.10 (-0.51, 0.30) | 0.35 (-0.08, 0.78) |  |  | 0.19 |
| β (95% CI), Model 3 | Reference | -0.17 (-0.58, 0.24) | 0.13 (-0.32, 0.57) |  |  | 0.76 |

*Generalized linear regression models were used to obtain coefficients for the change in memory Z-score for quintiles 2-5 versus the quintile 1 of energy intake from protein intake. Memory Z-score was calculated by summing the immediate and delayed recalls of 10 word list. The change in memory Z-score was computed as the score at baseline subtracting from that at follow-up.

^†^Model 1 was adjusted for age and gender.

^‡^Model 2 was adjusted for Model 1 plus education, urbanization, years of follow-up, smoking, alcohol intake, physical activity, memory Z-score, diabetes, BMI, systolic and diastolic blood pressure at baseline.

^§^Model 3 was adjusted for model 2 plus intake of energy, sodium, potassium, fat, and fiber.

**Table S3. Protein intake from different food sources and the change in subtraction Z-score***

|  | Consumption level | | | | |  |
| --- | --- | --- | --- | --- | --- | --- |
| Protein intake from  (% energy) | Quintile 1 | Quintile 2 | Quintile 3 | Quintile 4 | Quintile 5 | P-trend |
| All |  |  |  |  |  |  |
| Range | <11.36 | 11.36-12.86 | 12.86-14.27 | 14.28-16.15 | >16.15 |  |
| Participants | 617 | 616 | 616 | 617 | 617 |  |
| β (95% CI), Model 1 ^‡^ | Reference | 0.42 (0.21, 0.64) | 0.28 (0.06, 0.49) | 0.42 (0.21, 0.63) | 0.61 (0.40, 0.83) | <0.0001 |
| β (95% CI), Model 2 ^§^ | Reference | 0.27 (0.06, 0.48) | 0.15 (-0.06, 0.36) | 0.18 (-0.02, 0.39) | 0.31 (0.09, 0.52) | 0.0335 |
| β (95% CI), Model 3 ^¶^ | Reference | 0.20 (-0.01, 0.41) | 0.07 (-0.14, 0.29) | 0.10 (-0.11, 0.31) | 0.20 (-0.02, 0.41) | 0.26 |
| Plant foods |  |  |  |  |  |  |
| Range | <9.26 | 9.26-10.45 | 10.46-11.80 | 11.81-13.71 | >13.71 |  |
| Participants | 617 | 616 | 616 | 617 | 617 |  |
| β (95% CI), Model 1 | Reference | -0.38 (-0.59, -0.16) | -0.40 (-0.61, -0.18) | -0.46 (-0.68, -0.25) | -0.50 (-0.71, -0.28) | <0.0001 |
| β (95% CI), Model 2 | Reference | -0.14 (-0.35, 0.07) | -0.15 (-0.36, 0.06) | -0.14 (-0.36, 0.07) | -0.17 (-0.39, 0.04) | 0.16 |
| β (95% CI), Model 3 | Reference | -0.06 (-0.28, 0.15) | -0.09 (-0.31, 0.12) | -0.07 (-0.30, 0.15) | -0.12 (-0.34, 0.11) | 0.35 |
| Animal foods |  |  |  |  |  |  |
| Range | 0 | 0-0.88 | 0.88-2.07 | 2.08-4.17 | >4.17 |  |
| Participants | 745 | 488 | 616 | 617 | 617 |  |
| β (95% CI), Model 1 | Reference | 0.24 (0.03, 0.45) | 0.25 (0.05, 0.46) | 0.71 (0.50, 0.91) | 1.00 (0.79, 1.20) | <0.0001 |
| β (95% CI), Model 2 | Reference | 0.12 (-0.09, 0.33) | 0.11 (-0.09, 0.31) | 0.42 (0.22, 0.63) | 0.49 (0.26, 0.72) | <0.0001 |
| β (95% CI), Model 3 | Reference | 0.06 (-0.15, 0.27) | 0.00 (-0.20, 0.21) | 0.27 (0.05, 0.49) | 0.34 (0.09, 0.58) | 0.0039 |
| Grains |  |  |  |  |  |  |
| Range | <6.10 | 6.10-7.26 | 7.27-8.46 | 8.47-9.86 | >9.87 |  |
| Participants | 617 | 616 | 616 | 617 | 617 |  |
| β (95% CI), Model 1 | Reference | -0.25 (-0.46, -0.03) | -0.22 (-0.44, -0.01) | -0.62 (-0.84, -0.41) | -0.63 (-0.84, -0.42) | <0.0001 |
| β (95% CI), Model 2 | Reference | -0.05 (-0.26, 0.16) | 0.04 (-0.17, 0.25) | -0.32 (-0.53, -0.10) | -0.25 (-0.47, -0.03) | 0.0022 |
| β (95% CI), Model 3 | Reference | -0.04 (-0.25, 0.17) | 0.06 (-0.16, 0.28) | -0.29 (-0.52, -0.07) | -0.22 (-0.45, 0.02) | 0.0087 |
| Tubers |  |  |  |  |  |  |
| Range | 0 | 0-0.01 | 0.02-0.18 | 0.19-0.55 | >0.55 |  |
| Participants | 1208 | 25 | 616 | 617 | 617 |  |
| β (95% CI), Model 1 | Reference | 0.36 (-0.38, 1.10) | -0.03 (-0.21, 0.16) | 0.10 (-0.09, 0.28) | -0.19 (-0.38, -0.00) | 0.26 |
| β (95% CI), Model 2 | Reference | 0.35 (-0.37, 1.06) | -0.11 (-0.29, 0.07) | -0.01 (-0.19, 0.17) | -0.08 (-0.26, 0.10) | 0.40 |
| β (95% CI), Model 3 | Reference | 0.33 (-0.38, 1.04) | -0.13 (-0.31, 0.05) | -0.00 (-0.18, 0.18) | -0.02 (-0.20, 0.16) | 0.73 |
| Beans |  |  |  |  |  |  |
| Range | 0 | 0-0.59 | 0.60-1.64 | 1.65-3.32 | >3.32 |  |
| Participants | 776 | 457 | 616 | 617 | 617 |  |
| β (95% CI), Model 1 | Reference | 0.10 (-0.12, 0.32) | 0.08 (-0.12, 0.28) | 0.17 (-0.03, 0.37) | 0.18 (-0.03, 0.38) | 0.0624 |
| β (95% CI), Model 2 | Reference | 0.03 (-0.18, 0.25) | -0.01 (-0.21, 0.19) | 0.07 (-0.13, 0.26) | 0.09 (-0.10, 0.29) | 0.34 |
| β (95% CI), Model 3 | Reference | 0.02 (-0.19, 0.24) | -0.02 (-0.22, 0.17) | 0.03 (-0.17, 0.22) | 0.06 (-0.14, 0.25) | 0.60 |
| Vegetables |  |  |  |  |  |  |
| Range | <0.40 | 0.40-0.67 | 0.68-0.99 | 1.00-1.51 | >1.51 |  |
| Participants | 617 | 616 | 616 | 617 | 617 |  |
| β (95% CI), Model 1 | Reference | 0.10 (-0.12, 0.31) | 0.33 (0.12, 0.55) | 0.18 (-0.04, 0.39) | 0.19 (-0.02, 0.40) | 0.0606 |
| β (95% CI), Model 2 | Reference | -0.03 (-0.24, 0.18) | 0.22 (0.02, 0.43) | 0.10 (-0.10, 0.31) | 0.15 (-0.06, 0.35) | 0.0699 |
| β (95% CI), Model 3 | Reference | -0.04 (-0.24, 0.17) | 0.22 (0.02, 0.43) | 0.10 (-0.11, 0.31) | 0.20 (-0.02, 0.41) | 0.0305 |
| Fruits |  |  |  |  |  |  |
| Range | 0 | >0 |  |  |  |  |
| Participants | 2869 | 214 | . | . | . |  |
| β (95% CI), Model 1 | Reference | 0.84 (0.58, 1.10) |  |  |  | <0.0001 |
| β (95% CI), Model 2 | Reference | 0.40 (0.13, 0.66) |  |  |  | 0.0033 |
| β (95% CI), Model 3 | Reference | 0.37 (0.10, 0.63) |  |  |  | 0.0077 |
| Nuts |  |  |  |  |  |  |
| Range | 0 | >0 |  |  |  |  |
| Participants | 2854 | 229 | . | . | . |  |
| β (95% CI), Model 1 | Reference | 0.36 (0.10, 0.61) |  |  |  | 0.0063 |
| β (95% CI), Model 2 | Reference | 0.14 (-0.11, 0.39) |  |  |  | 0.28 |
| β (95% CI), Model 3 | Reference | 0.08 (-0.17, 0.33) |  |  |  | 0.53 |
| Red meat |  |  |  |  |  |  |
| Range | 0 | 0-0.35 | 0.36-1.08 | 1.09-2.30 | >2.30 |  |
| Participants | 1094 | 139 | 616 | 617 | 617 |  |
| β (95% CI), Model 1 | Reference | 0.01 (-0.33, 0.34) | 0.26 (0.07, 0.44) | 0.42 (0.23, 0.61) | 0.78 (0.59, 0.96) | <0.0001 |
| β (95% CI), Model 2 | Reference | -0.17 (-0.50, 0.16) | 0.12 (-0.07, 0.30) | 0.19 (-0.00, 0.38) | 0.36 (0.16, 0.56) | 0.0003 |
| β (95% CI), Model 3 | Reference | -0.27 (-0.60, 0.06) | 0.02 (-0.17, 0.20) | 0.06 (-0.13, 0.25) | 0.18 (-0.03, 0.39) | 0.0927 |
| Poultry |  |  |  |  |  |  |
| Range | 0 | >0 |  |  |  |  |
| Participants | 2850 | 233 |  |  |  |  |
| β (95% CI), Model 1 | Reference | 0.80 (0.54, 1.05) |  |  |  | <0.0001 |
| β (95% CI), Model 2 | Reference | 0.51 (0.26, 0.76) |  |  |  | 0.0001 |
| β (95% CI), Model 3 | Reference | 0.47 (0.22, 0.72) |  |  |  | 0.0003 |
| Fish/shrimp |  |  |  |  |  |  |
| Range | 0 | >0 |  |  |  |  |
| Participants | 2630 | 453 |  |  |  |  |
| β (95% CI), Model 1 | Reference | 0.80 (0.61, 0.99) |  |  |  | <0.0001 |
| β (95% CI), Model 2 | Reference | 0.44 (0.25, 0.63) |  |  |  | <0.0001 |
| β (95% CI), Model 3 | Reference | 0.42 (0.22, 0.63) |  |  |  | <0.0001 |
| Dairy |  |  |  |  |  |  |
| Range | 0 | >0 |  |  |  |  |
| Participants | 2757 | 326 |  |  |  |  |
| β (95% CI), Model 1 | Reference | 0.55 (0.34, 0.77) |  |  |  | <0.0001 |
| β (95% CI), Model 2 | Reference | 0.24 (0.02, 0.45) |  |  |  | 0.0320 |
| β (95% CI), Model 3 | Reference | 0.16 (-0.05, 0.38) |  |  |  | 0.14 |
| Eggs |  |  |  |  |  |  |
| Range | <0.17 | 0.18-0.86 | >0.86 |  |  |  |
| Participants | 1849 | 617 | 617 |  |  |  |
| β (95% CI), Model 1 | Reference | 0.44 (0.26, 0.61) | 0.55 (0.37, 0.72) |  |  | <0.0001 |
| β (95% CI), Model 2 | Reference | 0.22 (0.05, 0.40) | 0.16 (-0.02, 0.34) |  |  | 0.0306 |
| β (95% CI), Model 3 | Reference | 0.17 (-0.00, 0.35) | 0.07 (-0.12, 0.26) |  |  | 0.27 |

*Generalized linear regression models were used to obtain coefficients for the change in subtraction Z-score for quintiles 2-5 versus the quintile 1 of energy intake from protein intake. The change in subtraction Z-score was computed as the score at baseline subtracting from that at follow-up.

^†^Model 1 was adjusted for age and gender.

^‡^Model 2 was adjusted for Model 1 plus education, urbanization, years of follow-up, smoking, alcohol intake, physical activity, subtraction Z-score, diabetes, BMI, systolic and diastolic blood pressure at baseline.

^§^Model 3 was adjusted for model 2 plus intake of energy, sodium, potassium, fat, and fiber.

**Table S4. Protein intake from different food sources and the change in backward counting Z-score***

|  | Consumption level | | | | |  |
| --- | --- | --- | --- | --- | --- | --- |
| Protein intake from (% energy) | Quintile 1 | Quintile 2 | Quintile 3 | Quintile 4 | Quintile 5 | P-trend |
| All |  |  |  |  |  |  |
| Range | <11.36 | 11.36-12.86 | 12.86-14.27 | 14.28-16.15 | >16.15 |  |
| Participants | 617 | 616 | 616 | 617 | 617 |  |
| β (95% CI), Model 1 ^‡^ | Reference | 0.05 (-0.02, 0.11) | 0.06 (-0.01, 0.12) | -0.01 (-0.08, 0.05) | 0.03 (-0.04, 0.10) | 0.99 |
| β (95% CI), Model 2 ^§^ | Reference | 0.04 (-0.03, 0.10) | 0.05 (-0.02, 0.11) | -0.03 (-0.10, 0.03) | 0.01 (-0.06, 0.07) | 0.46 |
| β (95% CI), Model 3 ^¶^ | Reference | 0.03 (-0.03, 0.10) | 0.05 (-0.02, 0.12) | -0.03 (-0.10, 0.04) | 0.00 (-0.06, 0.07) | 0.43 |
| Plant foods |  |  |  |  |  |  |
| Range | <9.26 | 9.26-10.45 | 10.46-11.80 | 11.81-13.71 | >13.71 |  |
| Participants | 617 | 616 | 616 | 617 | 617 |  |
| β (95% CI), Model 1 | Reference | -0.03 (-0.09, 0.04) | -0.05 (-0.11, 0.02) | -0.04 (-0.10, 0.03) | -0.10 (-0.16, -0.03) | 0.0056 |
| β (95% CI), Model 2 | Reference | -0.02 (-0.08, 0.05) | -0.04 (-0.11, 0.03) | -0.02 (-0.09, 0.04) | -0.09 (-0.16, -0.02) | 0.0190 |
| β (95% CI), Model 3 | Reference | -0.01 (-0.08, 0.06) | -0.03 (-0.10, 0.03) | -0.02 (-0.09, 0.05) | -0.08 (-0.15, -0.01) | 0.0380 |
| Animal foods |  |  |  |  |  |  |
| Range | 0 | 0-0.88 | 0.88-2.07 | 2.08-4.17 | >4.17 |  |
| Participants | 745 | 488 | 616 | 617 | 617 |  |
| β (95% CI), Model 1 | Reference | 0.06 (-0.01, 0.12) | 0.01 (-0.06, 0.07) | 0.08 (0.02, 0.15) | 0.11 (0.04, 0.17) | 0.0007 |
| β (95% CI), Model 2 | Reference | 0.05 (-0.02, 0.11) | -0.00 (-0.07, 0.06) | 0.07 (0.01, 0.14) | 0.09 (0.02, 0.16) | 0.0136 |
| β (95% CI), Model 3 | Reference | 0.05 (-0.02, 0.12) | 0.00 (-0.06, 0.07) | 0.07 (0.00, 0.14) | 0.09 (0.01, 0.17) | 0.0298 |
| Grains |  |  |  |  |  |  |
| Range | <6.10 | 6.10-7.26 | 7.27-8.46 | 8.47-9.86 | >9.87 |  |
| Participants | 617 | 616 | 616 | 617 | 617 |  |
| β (95% CI), Model 1 | Reference | 0.02 (-0.05, 0.08) | -0.03 (-0.09, 0.04) | -0.06 (-0.13, 0.00) | -0.06 (-0.13, 0.00) | 0.0060 |
| β (95% CI), Model 2 | Reference | 0.03 (-0.04, 0.10) | -0.02 (-0.08, 0.05) | -0.05 (-0.12, 0.02) | -0.05 (-0.12, 0.02) | 0.0291 |
| β (95% CI), Model 3 | Reference | 0.03 (-0.04, 0.10) | -0.02 (-0.09, 0.05) | -0.05 (-0.13, 0.02) | -0.04 (-0.12, 0.03) | 0.0453 |
| Tubers |  |  |  |  |  |  |
| Range | 0 | 0-0.01 | 0.02-0.18 | 0.19-0.55 | >0.55 |  |
| Participants | 1208 | 25 | 616 | 617 | 617 |  |
| β (95% CI), Model 1 | Reference | 0.04 (-0.18, 0.27) | -0.01 (-0.06, 0.05) | 0.03 (-0.02, 0.09) | -0.02 (-0.08, 0.04) | 0.96 |
| β (95% CI), Model 2 | Reference | 0.04 (-0.18, 0.27) | -0.01 (-0.07, 0.04) | 0.03 (-0.03, 0.08) | -0.01 (-0.07, 0.05) | 0.97 |
| β (95% CI), Model 3 | Reference | 0.05 (-0.18, 0.28) | -0.02 (-0.07, 0.04) | 0.03 (-0.03, 0.08) | 0.00 (-0.06, 0.06) | 0.74 |
| Beans |  |  |  |  |  |  |
| Range | 0 | 0-0.59 | 0.60-1.64 | 1.65-3.32 | >3.32 |  |
| Participants | 776 | 457 | 616 | 617 | 617 |  |
| β (95% CI), Model 1 | Reference | -0.06 (-0.12, 0.01) | -0.03 (-0.09, 0.03) | -0.06 (-0.13, -0.00) | -0.04 (-0.10, 0.03) | 0.19 |
| β (95% CI), Model 2 | Reference | -0.06 (-0.13, 0.01) | -0.04 (-0.10, 0.03) | -0.07 (-0.14, -0.01) | -0.04 (-0.10, 0.02) | 0.11 |
| β (95% CI), Model 3 | Reference | -0.06 (-0.13, 0.01) | -0.04 (-0.10, 0.03) | -0.08 (-0.14, -0.01) | -0.04 (-0.10, 0.02) | 0.11 |
| Vegetables |  |  |  |  |  |  |
| Range | <0.40 | 0.40-0.67 | 0.68-0.99 | 1.00-1.51 | >1.51 |  |
| Participants | 617 | 616 | 616 | 617 | 617 |  |
| β (95% CI), Model 1 | Reference | 0.07 (0.01, 0.14) | 0.05 (-0.02, 0.11) | -0.00 (-0.07, 0.06) | 0.07 (0.01, 0.14) | 0.35 |
| β (95% CI), Model 2 | Reference | 0.07 (0.00, 0.13) | 0.04 (-0.03, 0.11) | -0.01 (-0.07, 0.06) | 0.07 (0.00, 0.13) | 0.39 |
| β (95% CI), Model 3 | Reference | 0.07 (0.00, 0.13) | 0.04 (-0.02, 0.11) | -0.00 (-0.07, 0.06) | 0.08 (0.01, 0.15) | 0.29 |
| Fruits |  |  |  |  |  |  |
| Range | 0 | >0 |  |  |  |  |
| Participants | 2869 | 214 | . | . | . |  |
| β (95% CI), Model 1 | Reference | 0.04 (-0.04, 0.12) |  |  |  | 0.34 |
| β (95% CI), Model 2 | Reference | 0.01 (-0.07, 0.10) |  |  |  | 0.79 |
| β (95% CI), Model 3 | Reference | -0.00 (-0.09, 0.09) |  |  |  | 1.00 |
| Nuts |  |  |  |  |  |  |
| Range | 0 | >0 |  |  |  |  |
| Participants | 2854 | 229 | . | . | . |  |
| β (95% CI), Model 1 | Reference | 0.02 (-0.06, 0.10) |  |  |  | 0.57 |
| β (95% CI), Model 2 | Reference | 0.01 (-0.07, 0.09) |  |  |  | 0.89 |
| β (95% CI), Model 3 | Reference | 0.01 (-0.07, 0.09) |  |  |  | 0.90 |
| Red meat |  |  |  |  |  |  |
| Range | 0 | 0-0.35 | 0.36-1.08 | 1.09-2.30 | >2.30 |  |
| Participants | 1094 | 139 | 616 | 617 | 617 |  |
| β (95% CI), Model 1 | Reference | 0.02 (-0.08, 0.13) | 0.01 (-0.05, 0.07) | 0.07 (0.02, 0.13) | 0.06 (-0.00, 0.12) | 0.0127 |
| β (95% CI), Model 2 | Reference | 0.01 (-0.09, 0.11) | 0.00 (-0.06, 0.06) | 0.06 (0.00, 0.12) | 0.04 (-0.03, 0.10) | 0.0886 |
| β (95% CI), Model 3 | Reference | 0.01 (-0.09, 0.12) | 0.00 (-0.06, 0.06) | 0.06 (0.00, 0.13) | 0.03 (-0.03, 0.10) | 0.13 |
| Poultry |  |  |  |  |  |  |
| Range | 0 | >0 |  |  |  |  |
| Participants | 2850 | 233 |  |  |  |  |
| β (95% CI), Model 1 | Reference | 0.07 (-0.01, 0.15) |  |  |  | 0.0863 |
| β (95% CI), Model 2 | Reference | 0.05 (-0.03, 0.13) |  |  |  | 0.21 |
| β (95% CI), Model 3 | Reference | 0.05 (-0.03, 0.13) |  |  |  | 0.26 |
| Fish/shrimp |  |  |  |  |  |  |
| Range | 0 | >0 |  |  |  |  |
| Participants | 2630 | 453 |  |  |  |  |
| β (95% CI), Model 1 | Reference | 0.08 (0.02, 0.14) |  |  |  | 0.0067 |
| β (95% CI), Model 2 | Reference | 0.06 (-0.00, 0.12) |  |  |  | 0.0504 |
| β (95% CI), Model 3 | Reference | 0.05 (-0.01, 0.12) |  |  |  | 0.11 |
| Dairy |  |  |  |  |  |  |
| Range | 0 | >0 |  |  |  |  |
| Participants | 2757 | 326 |  |  |  |  |
| β (95% CI), Model 1 | Reference | 0.03 (-0.04, 0.09) |  |  |  | 0.42 |
| β (95% CI), Model 2 | Reference | 0.01 (-0.06, 0.08) |  |  |  | 0.77 |
| β (95% CI), Model 3 | Reference | 0.01 (-0.06, 0.08) |  |  |  | 0.86 |
| Eggs |  |  |  |  |  |  |
| Range | <0.17 | 0.18-0.86 | >0.86 |  |  |  |
| Participants | 1849 | 617 | 617 |  |  |  |
| β (95% CI), Model 1 | Reference | 0.03 (-0.02, 0.09) | 0.04 (-0.02, 0.09) |  |  | 0.14 |
| β (95% CI), Model 2 | Reference | 0.02 (-0.04, 0.07) | 0.01 (-0.05, 0.07) |  |  | 0.62 |
| β (95% CI), Model 3 | Reference | 0.02 (-0.04, 0.07) | -0.00 (-0.06, 0.06) |  |  | 0.94 |

*Generalized linear regression models were used to obtain coefficients for the change in backward counting Z-score for quintiles 2-5 versus the quintile 1 of energy intake from protein intake. The change in backward counting Z-score was computed as the score at baseline subtracting from that at follow-up.

^†^Model 1 was adjusted for age and gender.

^‡^Model 2 was adjusted for Model 1 plus education, urbanization, years of follow-up, smoking, alcohol intake, physical activity, backward counting Z-score, diabetes, BMI, systolic and diastolic blood pressure at baseline.

^§^Model 3 was adjusted for model 2 plus intake of energy, sodium, potassium, fat, and fiber.

**Table S5. Protein intake from different sources and cognitive decline***

| Protein intake | Consumption level | | | | |  |
| --- | --- | --- | --- | --- | --- | --- |
| (% energy) | Quintile 1 | Quintile 2 | Quintile 3 | Quintile 4 | Quintile 5 | P-trend |
| All |  |  |  |  |  |  |
| Range | <11.36 | 11.36-12.86 | 12.86-14.27 | 14.28-16.15 | >16.15 |  |
| Events/Participants | 34/617 | 31/616 | 52/616 | 44/617 | 55/617 |  |
| OR (95% CI), Model 1^†^ | 1.00 | 0.88 (0.53-1.46) | 1.53 (0.98-2.40) | 1.30 (0.82-2.06) | 1.62 (1.04-2.53) | 0.0096 |
| OR (95% CI), Model 2^‡^ | 1.00 | 0.87 (0.52-1.45) | 1.57 (1.00-2.47) | 1.37 (0.86-2.20) | 1.72 (1.09-2.72) | 0.0037 |
| OR (95% CI), Model 3^§^ | 1.00 | 0.92 (0.55-1.55) | 1.66 (1.04-2.64) | 1.47 (0.91-2.38) | 1.87 (1.17-2.99) | 0.0015 |
| Plant foods |  |  |  |  |  |  |
| Range | <9.26 | 9.26-10.45 | 10.46-11.80 | 11.81-13.71 | >13.71 |  |
| Events/Participants | 35/617 | 45/616 | 40/616 | 46/617 | 50/617 |  |
| OR (95% CI), Model 1 | 1.00 | 1.32 (0.83-2.08) | 1.19 (0.74-1.90) | 1.35 (0.86-2.14) | 1.47 (0.94-2.31) | 0.12 |
| OR (95% CI), Model 2 | 1.00 | 1.40 (0.87-2.24) | 1.26 (0.78-2.04) | 1.43 (0.89-2.30) | 1.60 (1.00-2.56) | 0.0810 |
| OR (95% CI), Model 3 | 1.00 | 1.37 (0.85-2.21) | 1.24 (0.76-2.02) | 1.44 (0.88-2.36) | 1.62 (0.99-2.67) | 0.0781 |
| Animal foods |  |  |  |  |  |  |
| Range | 0 | 0-0.88 | 0.88-2.07 | 2.08-4.17 | >4.17 |  |
| Events/Participants | 55/745 | 25/488 | 48/616 | 43/617 | 45/617 |  |
| OR (95% CI), Model 1 | 1.00 | 0.67 (0.41-1.10) | 1.04 (0.69-1.56) | 0.94 (0.62-1.42) | 0.97 (0.64-1.47) | 0.80 |
| OR (95% CI), Model 2 | 1.00 | 0.68 (0.41-1.12) | 1.03 (0.68-1.57) | 0.93 (0.60-1.45) | 1.00 (0.62-1.62) | 0.77 |
| OR (95% CI), Model 3 | 1.00 | 0.68 (0.41-1.12) | 1.05 (0.69-1.60) | 1.00 (0.63-1.57) | 1.12 (0.67-1.85) | 0.48 |

*Logistic regression models were used to estimate ORs (95% CIs) for cognitive decline associated with protein intake from different sources. Cognitive decline was defined as the change in the composite cognitive score below the mean minus 1.5 standard deviations.

^†^Model 1 was adjusted for age and sex.

^‡^Model 2 was adjusted for Model 1 plus education, urbanization, years of follow-up, smoking, alcohol intake, physical activity, composite cognitive score, diabetes, BMI, systolic and diastolic blood pressure at baseline.

^§^Model 3 was adjusted for model 2 plus intake of energy, sodium, potassium, fat, and fiber.

**Table S6. The average protein intake from different sources of surveys completed until the first cognitive assessment and the change in composite cognitive score***

|  | Consumption level | | | | |  | Each 1% energy |
| --- | --- | --- | --- | --- | --- | --- | --- |
|  | Quintile 1 | Quintile 2 | Quintile 3 | Quintile 4 | Quintile 5 | P-value |  |
| All protein |  |  |  |  |  |  |  |
| Range | <12.19 | 12.20-13.22 | 13.23-14.17 | 14.17-15.55 | >15.55 |  |  |
| Participants | 617 | 616 | 616 | 617 | 617 |  |  |
| β (95% CI), Model 1^†^ | Reference | 0.06 (-0.04, 0.17) | -0.05 (-0.15, 0.06) | 0.04 (-0.06, 0.15) | 0.18 (0.08, 0.29) | 0.0037 | 0.03 (0.02, 0.05) |
| β (95% CI), Model 2^‡^ | Reference | 0.04 (-0.06, 0.14) | -0.09 (-0.19, 0.01) | -0.02 (-0.12, 0.09) | 0.03 (-0.07, 0.14) | 0.99 | 0.01 (-0.00, 0.02) |
| β (95% CI), Model 3^§^ | Reference | 0.04 (-0.06, 0.14) | -0.09 (-0.20, 0.01) | -0.03 (-0.14, 0.08) | -0.00 (-0.12, 0.11) | 0.49 | 0.01 (-0.01, 0.02) |
| Plant protein |  |  |  |  |  |  |  |
| Range | <9.31 | 9.31-10.43 | 10.44-11.28 | 11.28-12.31 | >12.31 |  |  |
| Participants | 617 | 616 | 616 | 617 | 617 |  |  |
| β (95% CI), Model 1 | Reference | -0.21 (-0.31, -0.11) | -0.22 (-0.33, -0.12) | -0.25 (-0.35, -0.14) | -0.33 (-0.44, -0.23) | <0.0001 | -0.05 (-0.06, -0.03) |
| β (95% CI), Model 2 | Reference | -0.14 (-0.24, -0.03) | -0.12 (-0.23, -0.02) | -0.14 (-0.25, -0.04) | -0.23 (-0.33, -0.12) | 0.0002 | -0.03 (-0.05, -0.01) |
| β (95% CI), Model 3 | Reference | -0.10 (-0.20, 0.01) | -0.08 (-0.19, 0.03) | -0.09 (-0.20, 0.02) | -0.17 (-0.28, -0.06) | 0.0113 | -0.02 (-0.04, -0.00) |
| Animal protein |  |  |  |  |  |  |  |
| Range | <0.87 | 0.87-1.95 | 1.96-3.28 | 3.29-5.09 | >5.09 |  |  |
| Participants | 617 | 615 | 617 | 617 | 617 |  |  |
| β (95% CI), Model 1 | Reference | 0.01 (-0.09, 0.12) | 0.13 (0.03, 0.24) | 0.19 (0.09, 0.30) | 0.36 (0.25, 0.46) | <0.0001 | 0.05 (0.04, 0.06) |
| β (95% CI), Model 2 | Reference | -0.03 (-0.13, 0.08) | 0.07 (-0.03, 0.17) | 0.11 (-0.00, 0.21) | 0.20 (0.08, 0.32) | 0.0002 | 0.03 (0.01, 0.04) |
| β (95% CI), Model 3 | Reference | -0.03 (-0.14, 0.07) | 0.05 (-0.06, 0.16) | 0.07 (-0.05, 0.20) | 0.14 (0.00, 0.28) | 0.0215 | 0.02 (0.01, 0.04) |

*Generalized linear regression models were used to obtain coefficients for the change in composite cognitive score for quintiles 2-5 versus the quintile 1 and per 1% increment in energy intake from protein intake. The change in composite cognitive score was computed as the score at baseline subtracting from that at follow-up.

^†^Model 1 was adjusted for age and sex.

^‡^Model 2 was adjusted for Model 1 plus education, urbanization, years of follow-up, smoking, alcohol intake, physical activity, composite cognitive score, diabetes, BMI, systolic and diastolic blood pressure at baseline.

^§^Model 3 was adjusted for model 2 plus intake of energy, sodium, potassium, fat, and fiber.
